# Implementation of a Surface Electromyography-Based Upper Extremity Exoskeleton Controller Using Learning from Demonstration

**DOI:** 10.3390/s18020467

**Published:** 2018-02-05

**Authors:** Ho Chit Siu, Ana M. Arenas, Tingxiao Sun, Leia A. Stirling

**Affiliations:** 1 Department of Aeronautics and Astronautics, Massachusetts Institute of Technology, 77 Massachusetts Ave, Cambridge, MA 02139, USA; tingxiao@mit.edu (T.S.); leia@mit.edu (L.A.S.); 2 Department of Mechanical Engineering, Massachusetts Institute of Technology, 77 Massachusetts Ave, Cambridge, MA 02139, USA; aarenas@mit.edu; 3 Institute for Medical Engineering and Science, Massachusetts Institute of Technology, 77 Massachusetts Ave, Cambridge, MA 02139, USA

**Keywords:** surface electromyography, learning from demonstration, exoskeletons, human experiments

## Abstract

Upper-extremity exoskeletons have demonstrated potential as augmentative, assistive, and rehabilitative devices. Typical control of upper-extremity exoskeletons have relied on switches, force/torque sensors, and surface electromyography (sEMG), but these systems are usually reactionary, and/or rely on entirely hand-tuned parameters. sEMG-based systems may be able to provide anticipatory control, since they interface directly with muscle signals, but typically require expert placement of sensors on muscle bodies. We present an implementation of an adaptive sEMG-based exoskeleton controller that learns a mapping between muscle activation and the desired system state during interaction with a user, generating a personalized sEMG feature classifier to allow for anticipatory control. This system is robust to novice placement of sEMG sensors, as well as subdermal muscle shifts. We validate this method with 18 subjects using a thumb exoskeleton to complete a book-placement task. This learning-from-demonstration system for exoskeleton control allows for very short training times, as well as the potential for improvement in intent recognition over time, and adaptation to physiological changes in the user, such as those due to fatigue.

## 1. Introduction

Upper-extremity exoskeletons have demonstrated the potential to augment the capabilities of human users [1,2], to assist disabled users with activities of daily living [3], and to be used as tools for rehabilitation following neurological disease or injury [4]. Current upper-extremity exoskeleton systems are often controlled by switches (e.g., [5,6,7,8]), pressure sensors (e.g., [9]), bend sensors (e.g., [10]), and similar mechanical inputs. Surface electromyography (sEMG) has been shown to be a potential control input for prosthetics [11,12,13,14], and exoskeletons (e.g., [15,16,17,18]), though these methods require precise placement of sensors over particular muscles, unless the muscles in question are relatively large, such as the biceps. sEMG has certain advantages over mechanical inputs, since sEMG signals of muscle contraction occur before the associated kinematics. Directly interfacing with muscle signals may mitigate the time lags associated with kinematic control and allow for more closely coordinated human-exoskeleton movement.

Control of upper-extremity devices is typically more difficult than control of lower-extremity exoskeletons, since the latter can rely on archetypical gait patterns, which do not have an upper-extremity analog. The degrees of freedom involved in common upper-extremity movements are also greater than those involved in most lower-extremity movements. Control of upper-extremity exoskeleton devices with sEMG will thus often use classification systems to reduce the complexity of the state space. sEMG classification systems have been demonstrated for in-air gesture recognition for teleoperation, sometimes in conjunction with inertial measurement (IMU) units. Wolf et al. [19] used this combination with a multiclass support vector machine to control a prosthetic hand and a mobile ground robot. Fukuda et al. [20] and Artemiadis and Kyriakopoulos [21] similarly used sEMG and IMU signals with a Gaussian mixture model to control a robotic arm based on the movement of the user’s arm. Supervised K-nearest neighbors classification was previously used by Zardoshti-Kermani et al. [22] for sEMG control of upper-extremity prosthesis. Other classification systems took advantage of the temporally-dependent nature of limb movements and used hidden Markov models as a classification method [23,24]. While these studies show potential for using sEMG for teleoperation and control of prostheses, with the exception of [24], they do not consider cases where the user is physically in contact with an object, and consequently do not consider cases where the human movement is affected by robot movement, both of which are elements needed for exoskeleton control. While [24] does involve sEMG classification during object contact, no exoskeleton is used in that study.

We aim to build on the work of these in-air systems to implement personalized sEMG control for upper-extremity exoskeletons, and take advantage of the sEMG signals preceding limb movement in order to improve fluency of human-exoskeleton movement. Beckers et al. [25] showed that anticipatory signals for grasp and release can be detected in sEMG data even with object contact during the gesture, but also found that slight shifts in the sensor placement—approximately 1 cm in any direction on the forearm—significantly change the anticipatory signals measured. A follow-on study using the same data [24] showed that a hidden Markov model classifier could correctly classify grasp and release 76% of the time, even when training on data from sensors that were periodically shifted up to 1 cm to simulate donning and doffing. These studies show that anticipatory sEMG control may be feasible with non-specific sensor placement, and may achieve even higher classification accuracy when sensor alignments are fixed.

In contrast to sEMG inputs provided by in-air gestures, there is a particular paradox inherent in using sEMG inputs to control exoskeletons that raises a unique challenge. For the most fluent movement, sEMG inputs for exoskeletons should be collected from the muscles of the limb that are coupled to the exoskeleton. However, if movement of a limb is constrained by an exoskeleton, the muscles of that limb may not be able to express the range of sEMG signals that are necessary for the desired movement, or the motor strategy itself may be adapted to the constrained movement. This situation means that a constrained limb cannot appropriately train an sEMG-based exoskeleton if it cannot move the exoskeleton, but the exoskeleton cannot move appropriately if it has not been trained to understand sEMG inputs.

One way to address this paradox was developed by Hamaya et al. [17,18], who used reinforcement learning to learn control policy parameters for an sEMG mapping from sensors at the biceps and triceps to a one-degree-of-freedom elbow exoskeleton. This method required a system identification step involving random perturbations applied by the exoskeleton on the human while the human attempted a lifting task, and an additional step where the system applied no torque while the user attempted the same task. The learned policy was then able to apply an appropriate torque to minimize sEMG activation when the user later attempted the lifting task. While this method has the advantage of mapping well to a continuous control output (exoskeleton torque), the need for both a system identification step and an untorqued step may make it difficult to scale the method up to exoskeletons with many degrees of freedom and dynamics that may be very different from the human limbs to which they are coupled. With a multi-DOF exoskeleton, the number of random perturbations needed for system identification grow significantly and the proposed method would become increasingly unwieldy for the user.

Kiguchi et al. [26] present an alternative method for using an adaptive controller to learn subject-specific sEMG signals for an elbow-shoulder exoskeleton. Here, a hierarchical control scheme was used that takes into account both sEMG and wrist force information. In this case, the sEMG mapping to motion is initialized using a fuzzy-logic-based artificial neural network (ANN) generated from previous sEMG experiments, which required muscle-specific sEMG sensor placement. The mapping was adapted to new users over time via backpropagation on the ANN.

Robot learning from demonstration (LfD) is a related body of work that can inform another solution. In this framework, appropriate robot actions are learned from examples provided by a human teacher, typically in a form that is very similar to the robot action [27]. For example, one might demonstrate an example motion to teach motion, or an example sentence to teach sentence construction. Instead of treating physical human-robot interaction as a disturbance, control systems utilizing LfD may instead learn from these interactions and correct the existing robot objective function [28]. Since highly personalized policies are required for sEMG control inputs [14], and there is tight physical coupling between the human “operator” and the exoskeleton “robot”, personalized LfD is an attractive option for learning a control mapping. This method also affords an opportunity to update appropriate sEMG to action mappings, even when the exoskeleton is in use. 

This paper builds on the sEMG-based anticipatory signal classification work of Siu et al. [24] to apply machine learning methods for exoskeleton control in a grasping task. We use an LfD method to train an anticipatory sEMG-based control system for exoskeletons, and implement and validate the method on a thumb exoskeleton with forearm sEMG inputs. The LfD used here solves the previously described control/actuation paradox by initially actuating the exoskeleton with a traditional limit-switch based controller before transitioning to sEMG-driven control.

There are several challenges faced by such a system. First, we use sEMG signals in a physically coupled human-robot system, where exoskeleton contact can change the signal. Second, since the system uses human-in-the-loop machine learning, care must be taken to ensure that the system acts appropriately even when it is being trained to avoid discomfort or injury. We implement novice (non-specific) placement of the sEMG sensors, since this is likely to occur when donning an exoskeleton in an operational setting as the muscles of the forearm can be difficult for a non-expert to palpate. In learning appropriate exoskeleton reactions, the system must also account for shifts in the muscles under the skin, which would occur even with expert sensor placement. In contrast to the work done by Hamaya et al. [17,18], our implementation on a thumb exoskeleton and forearm sEMG sensors must learn activations from smaller, more deeply embedded muscles of the forearm, as opposed to the larger surface muscles of the biceps and triceps. We also avoid using a random perturbation period for system identification, which removes a barrier to implementation on a many-DOF system. This paper presents the exoskeleton and algorithm used for the learning-from-demonstration sEMG controller, and an experiment to quantify the system performance. Specifically, we examine (1) the training time of the system; (2) the positive predictive value of the intent classification, and (3) whether or not the sEMG controllers anticipated the users’ movements.

## 2. Materials and Methods

### 2.1. System Description

**Figure 1:**
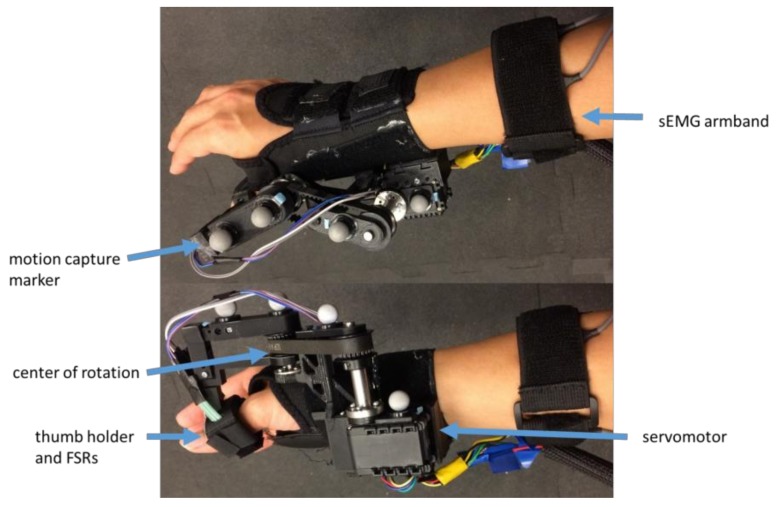
ILEXOS thumb exoskeleton from the top and side. Force-sensitive resistors (FSRs) are located on either side of the thumb (slightly offset from palmar and dorsal surfaces), between the user and the exoskeleton. The center of rotation is positioned near the carpometacarpal joint. Positioning of the entire exoskeleton as well as segment lengths can be adjusted via Velcro and set screws.

We present an exoskeleton control method that learns user intentions from interaction pressure between the user and the exoskeleton, along with and surface electromyography (sEMG) signals collected while the exoskeleton is in use. For this proof-of-concept, a single-degree-of-freedom thumb exoskeleton, the Interaction-based Learning Exoskeleton (ILEXOS Thumb) was used with the goal of assisting a user’s grasp and release motions (Figure 1). The system was not designed to offload the weight of the object grasped, support the full arm, or minimize muscle activity. The ILEXOS Thumb was developed to enable an examination of the accuracy and precision of the algorithm during the prescribed task. The exoskeleton used an off-the-shelf wrist brace (Mueller Sports Medicine, Praire du Sac, WI, USA) to which a servomotor (Dynamixel AX-12A, Robotis, Seoul, South Korea) was mounted using 3D printed and machined parts. The servomotor was connected to a thumb holder via a timing belt, with an adjustable center of rotation that was located over the carpometacarpal joint of the user.

Sensing was accomplished by two Flexiforce force-sensitive resistors (Tekscan, Boston, MA, USA) mounted on either side of the thumb, as well as a band comprised of six Delsys Bagnoli double-differential sEMG sensors located on the forearm (Delsys, Natick, MA, USA). The sEMG sensors were uniformly distributed along the band, and placed at the widest point of the forearm, without specific azimuthal placement of sensors on muscles. The force-sensitive resistors (FSRs) and sEMG sensors were connected to a single National Instruments USB-6351 Multifunction I/O device (National Instruments, Austin, TX, USA) which read inputs at 1000 Hz. The raw sEMG signals were processed with a 6th-order Butterworth filter (10–450 Hz), followed by a 60 Hz notch filter to remove noise from surrounding electrical equipment.

sEMG data were divided into 125 ms epochs for feature extraction, and successive epochs have 100 ms of overlap. This epoch size was chosen because it was between the 50 and 500 ms epochs used in literature [19,29,30,31], and is short enough to allow the features to be used as real-time control inputs. Fourteen sEMG features were extracted for each epoch and each sensor, for 84 real-valued control inputs per epoch. The features extracted were three binned frequency energies (low, medium, and high frequency) [32], three binned amplitude values (equally spaced between −1 and 1 mV [22]), mean average value, median frequency, max peak, number of slope sign changes, variance, Wilson amplitude (5 mV threshold), waveform length, and number of zero crossings [14].

Exoskeleton operation was segmented into a training phase and a test phase. During the training phase, a pressure-based limit switch controller was used. The test phase used one of three controllers: the pressure-based limit switch (LS), a non-adaptive EMG controller (NA), or an adaptive EMG controller (AC).

#### 2.1.1. Limit Switch Control (LS)

The pressure-based limit switch controller defaults the exoskeleton to an open grip, and closes the grip if sufficient pressure is applied to the interior FSR. The threshold for grip closure is adjusted on a person-by-person basis due to variations in thumb size and the way the exoskeleton is worn. sEMG data was collected along with pressure data during the phase, but was not used as a control input.

#### 2.1.2. Non-Adaptive EMG Control (NA)

The NA controller combines the pressure and sEMG data collected during the training phase to allow the exoskeleton to learn a user-specific correspondence between user intent and sEMG features via a supervised K-nearest-neighbors (KNN) classifier on fourteen signal features for each of the six sEMG sensors. Labeled data are generated by the user during a training period when the LS controller is used, with intent labels from the FSR pressure, and features from the sEMG. The collected sEMG features are standardized via mean-subtraction and divided by the feature values’ standard deviation, forming the model used for classification.

We use a supervised KNN for online learning of exoskeleton control because there is no model training time after data are collected, and it works well on small datasets. Both of these considerations are important for human-in-the-loop machine learning and control, where training time is limited by the patience and comfort of the user. The supervised KNN classifier is not the same as the more commonly used unsupervised KNN clustering algorithm, since the latter does not used labeled data [33].

To encourage the model to learn a classification that anticipates the motion of the user rather than simply replicating the reactive behavior of the limit switch controller, the labels for all features are shifted backwards in time by a single epoch. Thus, the label for epoch *t* is applied to the features from epoch *t* − 1, so that labels apply to the 125 ms time window that ended 25 ms before the original label time. The last epoch in a trial is assumed to have the same label as the epoch before it. The optimal amount of epoch shift depends on the length of each epoch and amount of time between successive epochs, and the single-epoch shift used here was chosen after preliminary experiments using zero to six epochs of shifting.

Unlabeled data during the test phase were classified by comparing new data points to its K nearest neighbors. We set K to be 50, and the distance to neighbors was the Euclidean distance in feature space. Each of the 50 neighbors adds a weight equal to one over the Euclidean distance to its label, and the label with the greatest weighted sum is considered the label of the new data point. With this controller, up to 1500 data points from the training data are used to form the KNN model. If the training data contains more than 1500 data points, 1500 points are randomly sampled from the data. The number of data points was capped at 1500 to maintain real-time control using the selected system software and hardware.

When this controller is in use, pressure data continued to be collected for post-hoc analysis, though only the sEMG data were used as control inputs within the test phase. The sEMG-to-actuation mapping used in this controller remained constant for all trials.

#### 2.1.3. Adaptive EMG Control (AC)

The AC controller was initialized in the same way as the NA controller. This initialization means that the first test trial using either the AC or the NA controllers is equivalent (though different mappings may be learned depending on the associated training). This controller diverges from the NA controller after the first test trial.

Between test trials, the AC controller performs an update to the sEMG mapping via the KNN classifier. First, epochs from the preceding test trial were labeled. A “close” label was applied if the pressure on the interior FSR exceeded that of the exterior FSR by the threshold set for LS control. An “open” label was applied if the pressure differential exceeded the threshold in the opposite direction. If neither threshold is exceeded, the open/close actuation command previously given for the epoch is assumed correct and the corresponding label is applied.

Next, a “full KNN model” is created using all the data collected during the previous AC trials, along with the training trial. Since KNN runtime scales linearly with the number of data points, using the full model is prohibitively expensive for real-time exoskeleton operation. We thus construct a reduced model with fewer data points that is as close to the full model as possible, as measured by the L2 norm between the models. A reduced model consisting of 1500 randomly sampled points from the previous trial is built, and the L2 norm between this and the full KNN model was calculated.

Since calculating the L2 norm via integration is infeasible on the 84-dimensional feature space, we use a Monte Carlo integration of the norm with importance sampling [34] to emphasize the feature space where the user is providing inputs. Consider P the set of 1500 points used to build the reduced model. We use a two-step importance sampling method by sampling points x from P and then sampling again from the space near the points x. Specifically, Monte Carlo samples are chosen by repeatedly resampling x from P and then sampling from a normal distribution $N\left(x,\sigma_{NN}\right)$ where $\sigma_{NN}$ is the square root of the mean distance between nearest neighbors in P.

Reduced models are constructed and evaluated via random resampling of new sets P until a predetermined maximum time was reached (two minutes for the current experiment, the time subjects are allowed to rest between trials), and the model with the lowest L2 norm was kept and used for the next trial. This process allows a new KNN model to be generated after each trial that has increased influence from the sEMG data provided in the most recent trial. The exoskeleton’s mapping of intent to action thus changes over time to reflect any changes in the subject’s intent expression.

### 2.2. System Operation

A typical concept of operations for training and using the sEMG control mode is described as follows:

1. *System fit.* The user dons the sEMG armband and the exoskeleton, and adjustments are made to the thumb holder and actuator placement to ensure comfort and an aligned center of rotation.
2. *sEMG calibration.* The user holds his or her arm steady, laterally in his or her lap, while the armband records five seconds of sEMG data, in order to establish a nominal resting mean and standard deviation for each sensor.
3. *Range-of-motion calibration.* The user moves the unpowered exoskeleton to acceptable “close” and “open” positions for the desired task, which are then recorded by the exoskeleton.
4. *Limit switch deadband calibration.* A deadband for the limit switch of the pressure-controlled case is set for the individual.
5. *Training.* The LS mode is used initially to allow the user to familiarize themselves with using the exoskeleton, and then the desired task is performed.
6. *Testing.* If the NA or AC mode is being used, the system is switched to an sEMG-controlled mode, which uses a classification model based on previous data to move the exoskeleton according to the inferred user intent. If the LS mode is being used, then the controller remains the same as it was in training.
7. *Updates.* If the AC mode is being used, the system periodically updates its sEMG mapping. For the experiment (described below), these updates occur during breaks in the task.

### 2.3. System Validation Experiment

The efficacy of the learned sEMG control system was evaluated using a tabletop book-shelving task. Twenty people were consented for the study, but two subjects’ data were unusable due to technical issues with the exoskeleton in one case, and unforeseen distractions for the subject in another. For one participant, data from two out of the twelve total test trials was unusable as the exoskeleton had a wire that became disconnected and data were not captured. The remaining trials for this subject were appropriately captured and are included in the analysis. 

The 18 participants that completed the study and were included in data analysis consisted of eleven males and seven females, all of whom were right-handed, had 20/20 or corrected to 20/20 vision, and had no self-reported disabilities or injuries affecting the use of their right arms. The experiment protocol was approved by the MIT Committee on the Use of Humans as Experimental Subjects (protocol 1454402), and all participants provided written informed consent. All participants received up to $20 as compensation. Subject age ranges and collected anthropometry data are shown in Table 1.

**Figure 2:**
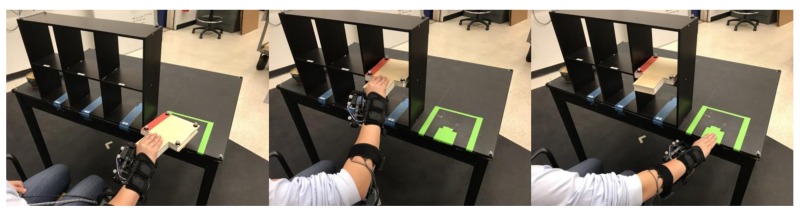
Grasping action taken by subjects. For each motion, the subject grasps the book from the starting position on the table (**left**), places the book onto a cubby (**middle**) and the taps the marked spot on the table (**right**) before reversing the series of actions and placing the book back onto the starting position. For each grasp, the thumb is on the bottom supporting the book so that thumb closure is required for the grip.

During the experiment, participants were seated in front of a tabletop bookshelf with six shelves (70 cm wide by 41.5 cm high by 15 cm deep), and asked to move a book between the tabletop and shelves on the bookshelf using their right hands while wearing the exoskeleton. Participants were instructed to move the book in a palm-down position, using their thumbs to support the bottom of the book (Figure 2). Two corners of the book were cut out to allow for better motion tracking of the subjects’ thumbs during the task. Requiring the thumb to be on the bottom limited finger flexion/extension during the grasping task, and limited participants from using their fingers to compensate for altered thumb movement due to the exoskeleton. Lateral variation in the placement locations of the book created increased potential for subdermal muscle shifts, for which the sEMG controllers must account. This task permitted an evaluation of the timing of the thumb assistance while limiting the compensatory motions a user could perform during the task, thus enabling validation of the system’s behavior.

A single iteration of the task consisted of grasping a book on the tabletop, placing it in one of six locations on the bookshelf, releasing the book, touching the table where the book was originally placed, and then grasping and moving the book back to its original location (Figure 2).

Steps 1 through 4 of the concept of operations were followed at the beginning of the experiment to adjust the system for each user. The experiment itself consisted of three blocks, one to test each controller. After the system adjustment steps at the beginning of the experiment, each block began with a single-trial training phase consisting of six iterations of the task, once per location available on the bookshelf. The training period was always performed with the limit switch controller (step 5 of the concept of operations). This training period was not set at a fixed time duration, but rather ended when a user finished one set of six motions, utilizing all the spots on the shelf. There is thus some variability in training time, as well as the amount of data and the proportion of the data that is labeled as grasp or release.

During the subsequent test phase, subjects performed four trials, each consisting of twenty-four iterations of the task, where each of the six locations on the bookshelf was used four times (for a total of 96 iterations of the task during the test phase of each block). These test phases were performed with the LS, NA, or AC controllers, depending on the block (step 6), with the subjects unaware of the nature of the controllers they were using. Trials were separated by a two-minute break and blocks were separated by five-minute breaks to allow the subject to rest. Subjects took Likert scale and NASA Task Load Index [35] surveys during those two- and five-minute breaks (results for the surveys are not reported in this paper). During the AC block, the two minutes were also used to update the sEMG model (step 7). Three block orders were used to control for participant learning effects, while enabling an opportunity to examine the effect of order. The block orders were (FB, NA, AC), (FB, AC, NA) and (AC, NA, FB), where each one was used with six subjects.

For the purpose of these comparisons, a grasping action (thumb close) is considered a positive result (e.g., true positive or false positive), and a release (thumb open) is a negative result (e.g., true negative or false negative). Ground truth was determined by the pressure on the FSR sensors, which should be high when a subject is holding the book, regardless of the controller used, due to the nature of the grasp being performed. Thus, a true positive occurred when the controller actuates to a close position when the user has a high pressure signal on the palmar thumb FSR (beyond the pre-set activation threshold for the LS controller). A false positive occurred when the controller actuated to a close position when the user did not have a high palmar thumb FSR signal, and continues to not have a high signal for the duration of the close actuation. Note that this scheme for obtaining ground truth means that false positives cannot occur with the limit switch controller by definition, since it uses the pressure signal directly for actuation. This setup also allows us to use the limit switch controller as a baseline against which to compare the other controllers. 

### 2.4. Data Analysis

Data analysis for this paper focuses on examining three aspects of the exoskeleton behavior that influence system usability: (1) the training time of the system; (2) the precision (positive predictive value) of the EMG intent classification; and (3) whether or not the EMG controllers anticipated the human.

The training times were calculated as the time between the first exoskeleton actuation command to either the last actuation command or the end of the trial (whichever came first). The times were normally distributed according to a Shapiro-Wilk normality test (*p* < 0.05), so a one-way analysis of variance (ANOVA) was used to find differences in the times between controllers.

The precision of the sEMG-based prediction was evaluated in two ways—(1) the number of true and false positives across trials and (2) the duration of the positive responses. Recall, a positive response is defined to be when the system is in a closed state. We evaluate precision (true/false positive responses) because ground truth information on negative predictions (i.e., when the user did not intend to grasp) is harder to infer. The grasping pressure provides a much clearer signal than the equivalent pressure signal when the intent is *not* to grasp. The duration of these positives is important because it acts as a measure of how useful a true positive was (if it lasted the duration of an intended grasp) or how detrimental a false positive was (how long the user was stopped from doing what he or she intended). Both the number and duration of positive responses were determined to be non-normal using the Shapiro-Wilk test (*p* > 0.05), so nonparametric Kruskal-Wallis tests were used. 

An initial Kruskal-Wallis test was performed for each combination of controller and response type, with an independent variable of trial (6 tests in total). All tests for trial were non-significant for both number and duration of grasps (*p* > 0.05), thus trial data were pooled to examine the other independent variables. As the Kruskal-Wallis test does not include interaction effects in the omnibus test, an independent variable was defined to enable examination of the individual treatments. For the number of positive responses and action duration, the independent variable defined included controller and response type (e.g., AC true, NA false, etc.), with trials pooled as previously described. Omnibus Kruskal-Wallis tests were then performed for both number and duration, and Dwass-Steel-Critchlow-Fligner post-hoc pairwise comparisons were performed if the corresponding omnibus test was significant, enabling underlying interactions to be examined.

**Figure 3:**
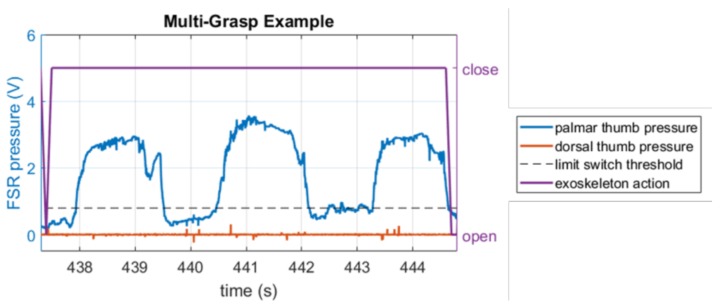
Example of a “multi-grasp” where the palmar thumb pressure crosses the limit switch threshold from below multiple times within a single exoskeleton close action, with below-threshold periods longer than 0.1 s interspersed.

A special case of “multi-grasp” was also considered in the analysis of precision. A multi-grasp refers to a scenario within a single close-open cycle of exoskeleton movement where the pressure profile indicates multiple grasps. Using the pressure profile, the multi-grasp was defined to occur if the pressure signal crosses the limit switch threshold from below more than once and remained below the threshold for more than 0.1 s (one servo control cycle) between crossings (Figure 3). These instances involve elements of both true positives (the initial close) as well as false positives (a continued close state when the pressure drops below the threshold). Since these are not as easily classified, we remove these occurrences from the true and false positive analysis and examine them separately.

**Figure 4:**
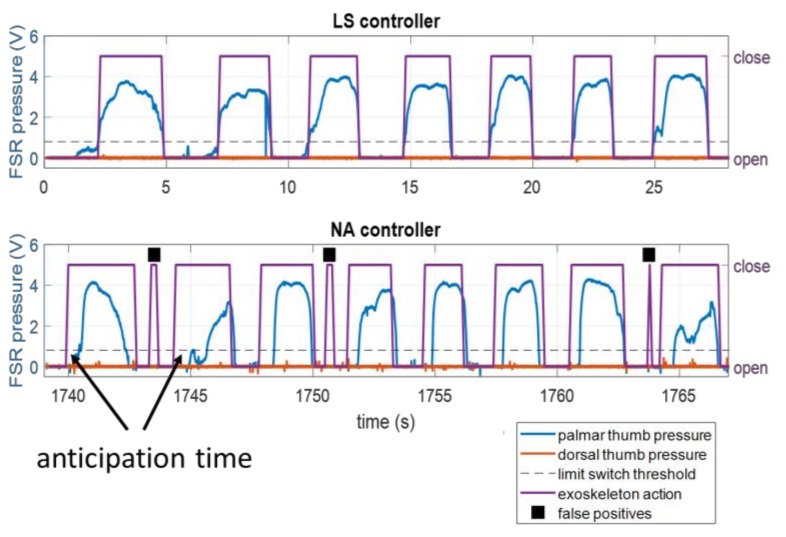
Example of thumb pressures detected during operation of a limit switch controller (**top**) and an EMG controller (**bottom**) along with exoskeleton action. The thumb pressure and limit switch threshold lines correspond to the left axis, and the exoskeleton action corresponds to the right axis. The bottom plot shows how exoskeleton actuation often occurs before the limit switch threshold is reached with the EMG controller, as the black line changes before the red line hits the dashed limit. False positive predictions are marked with black squares.

In order to quantify anticipatory exoskeleton behavior, we define the *anticipation time* for a true positive grasp as the time between the start of an EMG-triggered grasping motion and the time when the pressure threshold is crossed (Figure 4). Since anticipation time is necessarily zero for the LS controller, only the times associated with NA and AC were compared. The Shapiro-Wilk test supported that the anticipation times were non-normal, so the sign test was used to test whether each controller’s times were significantly different from zero, and the Wilcoxon rank-sum test was used to determine whether or not each controller’s times were significantly different from each other.

## 3. Results

### 3.1. Training Time

**Figure 5:**
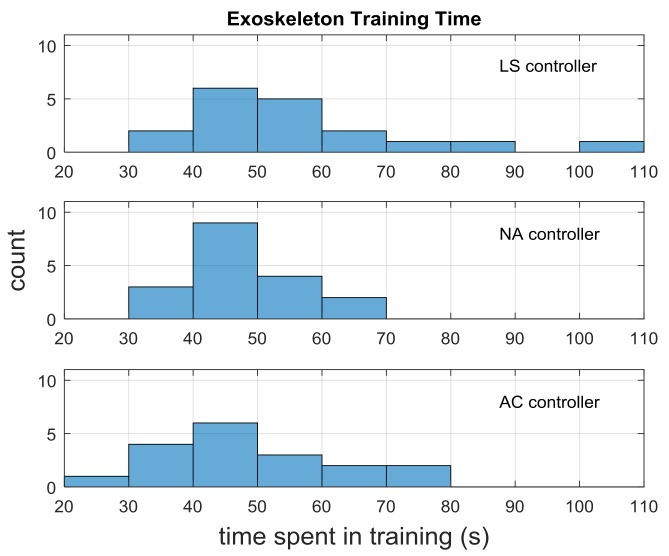
Distribution of the time spent completing the six-motion training task across all subjects. The mean and standard deviation were 51 s and 14 s respectively.

Figure 5 shows the distribution of times spent in training for each of the three controllers. The training time shown here is the time between the start of the first grasp and the end of the last grasp. There are a few additional seconds of time when data are still being collected before and after this period, but this definition is the one that is operationally relevant to the user, since that is when the user is actively training the system. Training occurs before the LS test trials for consistency with the other blocks, though data collected at that time is not used during the experiment.

The mean training time for the three controllers is not significantly different (F(2, 51) = 1.92, *p* > 0.05), and the mean time across all cases is 51 ± 14 s. Thus, users have similar exposure to the system within each training period.

### 3.2. Precision of Prediction

**Figure 6:**
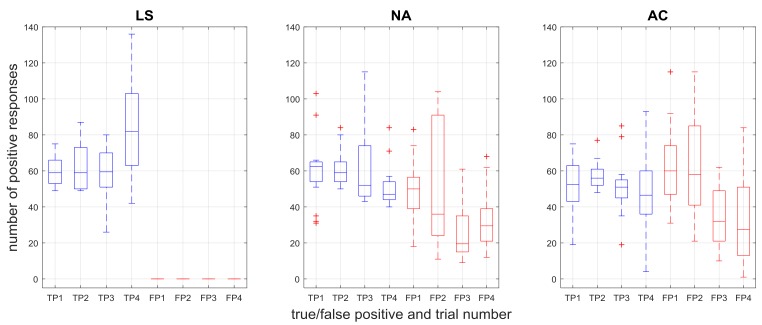
Number of true positive (TP) and false positive (FP) responses across test trials for the three controllers.

**Figure 7:**
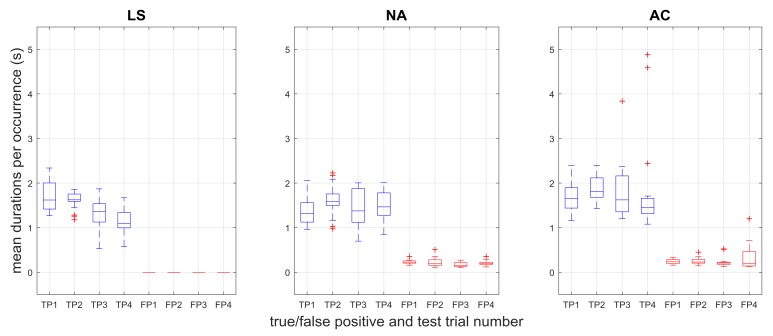
Mean duration (seconds) of true positive (TP) and false positive (FP) responses across test trials for the three controllers. Significant differences (*p* < 0.01) were found between true and false positive durations within each controller. Multi-grasps were removed in the data shown here.

The number of true and false positives across trials for each controller is shown in Figure 6, and the duration of true and false positives is shown in Figure 7. The limit switch controller exhibits no false positives (both figures, LS plots), since the pressures that directly drive the LS controller are used as the ground truth for intent.

The Kruskal-Wallis tests with an independent variable of controller crossed with response found significant effects for both the number of responses (H = 221.918, *p* < 0.01), and the response time (H = 362.080, *p* < 0.01). For the number of responses, the median number of true positives for LS was significantly greater than both NA (*p* < 0.01) and AC (*p* < 0.01). There was no significant difference in the median number of true positive responses between NA and AC. The median number of false positives was significantly greater for AC than NA (*p* = 0.045). The median number of true positives was significantly different from false positives for NA (*p* < 0.001), although was not significantly different for AC (*p* > 0.05). For the median duration, there was no significant difference between the median true positive durations for LS and NA (*p* > 0.05) or LS and AC (*p* > 0.05). The duration of true positives was found to be greater than the duration of false positives for all cases (*p* < 0.05). 

### 3.3. Occurrence of Multi-Grasps

**Figure 8:**
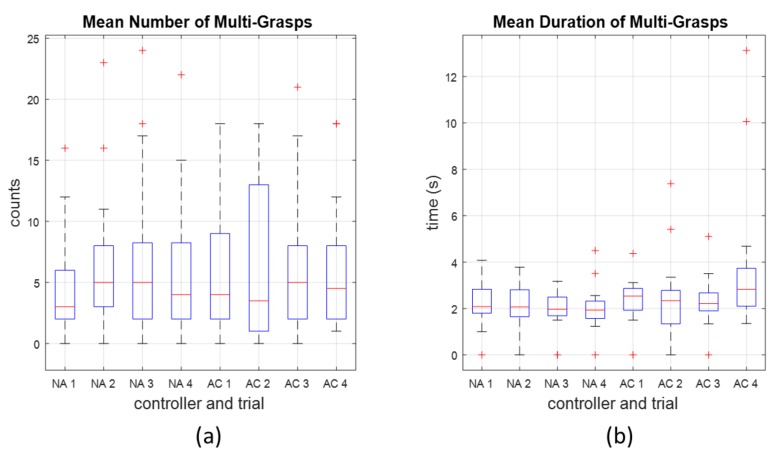
Number (**a**) and duration (**b**) of multi-grasps in the trials using NA and AC controllers. Extreme outliers in terms of duration occurred for two subjects during the fourth trial of the AC controller.

As previously stated, we removed occurrences of “multi-grasps” from our precision analysis, and considered them separately. Figure 3 showed an example of a multi-grasp, where a single exoskeleton close action was accompanied by three distinct grasps in the pressure profile. Figure 8 shows the number and duration of multi-grasps across trials. We specifically note two subjects for which the mean multi-grasp durations were outliers during their last AC trial (Figure 8a, right-most box plot). For these subjects, the exoskeleton remained closed for long periods, and they were unable to command it to open, but proceeded with the task anyway. From observation, we saw that apart from these outliers, many of the other examples of multi-grasps were two-grasp periods that resulted when subjects moved the book from the shelf to the table, and released slightly before re-grasping, but did so very quickly without allowing the exoskeleton to actually open during this period. For these multi-grasps, it was unclear whether the subjects intended to command the exoskeleton to open or not, but the lack of exoskeleton motion here did not hinder the task.

### 3.4. Grasp Durations

**Figure 9:**
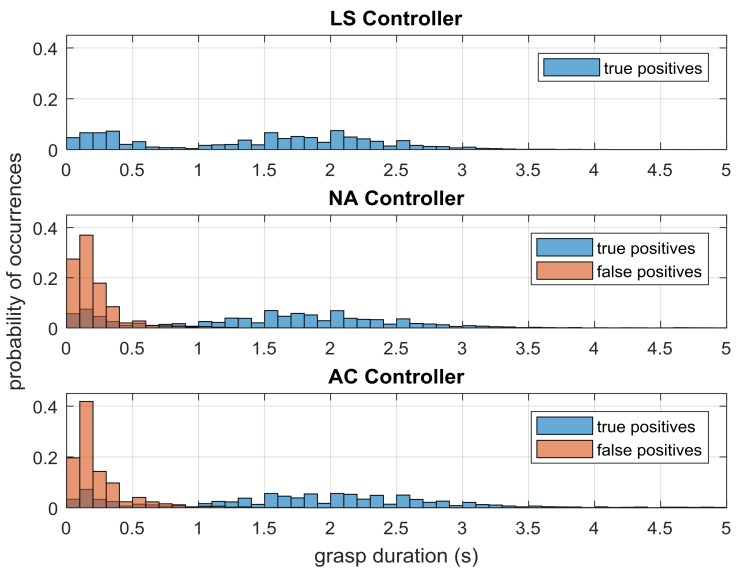
Histogram of positive response times shows that true positives are bimodal and that the shortest actuation durations are dominated by false positives. Only grasps shorter than 5 s are shown, but grasps that were longer than 5 s occurred for LS (*n* = 7), NA (*n* = 1), and AC (*n* = 24) controllers.

The distribution of the grasp durations is shown in Figure 9, which pools all of the grasps that were detected throughout the test trials for the entire experiment. For all three controllers, the distribution of true positive responses is bimodal, with a cluster at approximately 0.25 s, and another at approximately 2 s. Since this distribution occurs even with the limit switch controller, the short-duration cluster likely represents inadvertent actuation of the exoskeleton by the subject when briefly pressing up against the pressure sensor, while the longer-duration cluster likely represents periods when the user is actually grasping the book. Notably, the NA and AC controllers show that a large number of the false positives occur for no more than 0.2 s, which represents one to two control cycles for the exoskeleton, and approximately 5 degrees of movement.

### 3.5. Anticipating User Movement

In the non-anticipatory case (the limit switch), actuation occurs as a reactive measure when the pressure of the user’s thumb crosses a given threshold. Part of the purpose of ILEXOS was to reduce the movement delay and the need for constant pressure by the user with an anticipatory sEMG control scheme. Figure 4 was a representative comparison of the exoskeleton sensing and actuation taken during one subject’s use of the LS and the NA controllers. In the LS case (top), the exoskeleton movement in black is triggered exactly as the user’s pressure on the palmar sensor crosses the limit switch threshold. In the NA case (bottom), the shape of the pressure curve still roughly corresponds to the exoskeleton behavior (higher pressure during grasp, which would always be the case due to contact with the book), but the actuation for grasp occurs before the pressure threshold is passed. Similar behavior is seen throughout the NA and AC controller trials.

**Figure 10:**
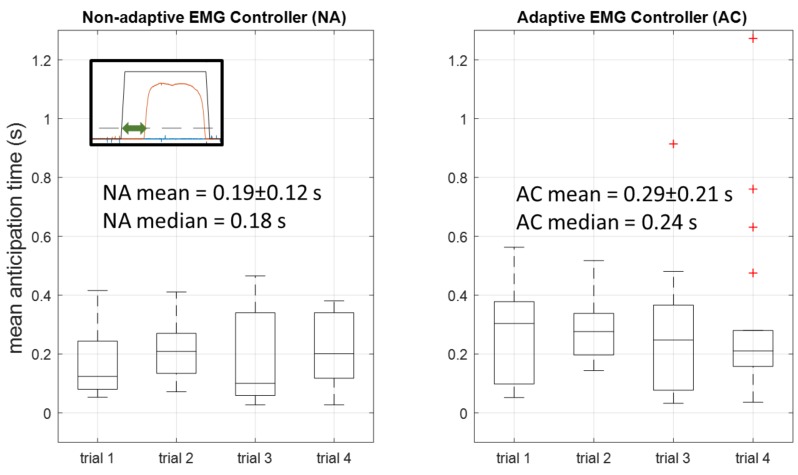
Mean and median anticipation time (seconds) of true positive grasps. Anticipation time for a given grasp is calculated as the time that the grasping pressure exceeds the trigger threshold minus the time that a grasp was triggered by the EMG controller, as shown by the green arrow on the inset example.

Figure 10 shows the mean anticipation times of the NA and AC by controller and across trials. Sign tests for the anticipation times with a predictor of controller type showed a significant difference of each controller’s anticipation times from a time of zero (W = 70, *p* < 0.01 for NA; W = 72, *p* < 0.01 for AC). The Wilcoxon Rank-Sum test supported a significant difference between the anticipation times for the two controllers (z = −2.86, *p* < 0.01).

## 4. Discussion

The results show that personalized sEMG-based exoskeleton control is possible with machine learning for this grasping task on a 1-DOF exoskeleton with short training periods. The learning from demonstration scheme implemented in ILEXOS allowed us to use a traditional limit switch controller to learn sEMG inputs, and then improve on that performance by anticipating the user by time-shifting the intent labels collected during the limit switch phase. The learned mappings are robust to both novice sensor placement and to subdermal muscle shifts. We specifically assessed three elements of the experiment: the training time of the system, the precision of the EMG intent classification, and the system’s ability to anticipate the user’s movements.

### 4.1. Training Time

The training time of our system was based on completing twelve placement motions on six shelf locations, and took approximately one minute for a novice user to complete. During this training time the exoskeleton moved in response to the user, but was controlled via a pressure-based limit-switch. This training time is on par with times used in other upper-extremity sEMG classification experiments, such as the two minutes used by Chan and Englehart [23] in their in-air gesture-recognition (non-exoskeleton) study, or the one minute used by Hamaya et al. [17] in their one-DOF elbow exoskeleton study.

Comparing training times between studies is not straightforward, since many elements of these studies (in-air vs. object contact, exoskeleton vs. hand gesture, number of classification labels, accuracy, sensor placement, number of subjects, etc.) are highly variable. However, shorter training time is certainly operationally desirable, as is robustness to imprecise sensor placement. In an industrial setting, for example, the ability to quickly don and begin using an augmentative system could translate to higher rates of adoption and lower user frustration in comparison to systems requiring significant training time for the classifier (to understand intent) and/or for the user (to precisely place sensors).

### 4.2. Precision of Prediction

It is promising to see that the duration of responses between true and false positives were significantly different in each case but that true positive durations in both sEMG-controlled cases were not different from the LS case (Figure 7). These results show that as far as grasp duration, the positive response performance of the three controllers is comparable, which is a necessary result to show in order to have sEMG-based control be at least as useful as limit switch methods.

On the other hand, although the duration results show that the ILEXOS Thumb is usable under sEMG-controlled conditions, one potential issue is the relatively large *number* of false positives. Figure 6 showed that the NA and AC controllers have false positive counts that are comparable to the number of true positives (though most of these are very short, as shown in Figure 9). One factor driving the presence of false positives is the specific training procedure and the underlying task selection. In our task, subjects tap the book’s starting location on the table after putting the book on the shelf. This interim tapping step was included as a way to provide examples within the time series where the subjects’ arms were on the right side of the table, but they were not grasping. Our pilot testing found without such examples, the exoskeleton would begin a grasp whenever the subject had his or her hand on the right, regardless of whether or not a grasp was intended.

While the tapping motion did remove this confusion from the system, it introduced a signal that was similar to the sEMG signal of a grasp if the subject was tapping hard. This was observed in many cases with both sEMG controllers, where tapping the table induced a short actuation by the exoskeleton, likely resulting in many of the 0.1 and 0.2-s false positives seen in Figure 9. Since subjects tap the table once for every two grasps that they perform, it is possible that up to half of the false positive counts recorded in this experiment were the result of these tap-induced motions.

Such false positives might be removed with additional training time or different AC controller updates assumptions (such as removing short-duration close labels from updates). However, the short duration of these motions means that they are largely operationally insignificant, though the presence of such unintended motions may affect the user’s trust in the system. On the other hand, such false positives could present a proprioceptive feedback to help the user learn the system’s behavior and adjust their own inputs as a kind of error augmentation, though such false positives would have to be limited to prevent user frustration or injury. 

We also considered the occurrence of multi-grasps—multiple grasps as indicated by pressure within a single period of exoskeleton close action. These occurrences had elements of true positive, false positive, and false negative (not opening when it should) responses, and were considered separately. In some cases, these were simply the result of the participant acting very quickly in letting go of and re-grasping the book when it was placed on the table, which may be an indication of a change in grasping strategy. These particular multi-grasps would likely have been reduced or eliminated with more explicit instructions to the participants. Although these grasps were not observed to be affecting the performance of the task.

However, we do note two specific cases of the AC treatment where multi-grasps were exceedingly long, and characterized by a near-complete inability of the user to command the exoskeleton to open during an entire trial (Figure 8). In these cases, it is likely that the combination of assumptions made by the AC controller update—that pressure in a given direction indicated intent to move in that direction *and* that lack of pressure meant that the label obtained from the sEMG classifier was correct—drove the classifier to be heavily biased towards a close label. It is unclear why this occurred with these two subjects, but not with others. That such a phenomenon could happen, at least with two of the subjects and specifically with the AC controller, points to the need for additional refinements of the AC updates in order to prevent this particular failure mode (described further in Section 4.3).

A hybrid approach to control using both sEMG and pressure at the same time could significantly improve prediction performance for an operational use case. This combination avoids the potential issue of the exoskeleton not moving when the subject is pressed up against one side because of a mismatch between pressure and sEMG labeling during a particular time, but keep the advantage of anticipatory signaling by starting exoskeleton movement when the associated sEMG signal is detected. We did not evaluate the hybrid controller in this study as we were specifically examining if an sEMG-only controller would enable fluent task performance.

### 4.3. Adaptive Controller Updates

The adaptive sEMG controller was intended as a way for the control system to adapt to sEMG changes over time, particularly those caused by user fatigue [36]. The AC mode had the potential additional benefit of gathering a larger dataset than the initial training period could provide to enable an improved model, but had the potential downside of being a changing control mapping that updated once every few minutes due to our experiment protocol. The changing model with this particular update rate may have been more difficult for the human to learn when compared to a static model, or one that continuously updated.

Qualitatively, subjects reported differences in experiences after test trial 1 with the AC controller. Some users reported improved exoskeleton performance, while two users encountered significant difficulty with multi-grasps in the later trials. While the design goal was for the system to be easier to operate as the additional data collected provides an ability to improve the controller, the dynamic change in the controller could add a challenge for the operator if the system does not change at an appropriate rate. Finding such an appropriate rate of change would take further work, and may depend on the task and/or the user. The underlying assumptions within the AC update may also be more appropriate for some operators than for others. The second assumption regarding the correctness of sEMG labels when pressure data was lacking may be inappropriate in some cases because subjects do not necessarily oppose the exoskeleton when it is behaving inappropriately, as may be seen in the constant near-zero dorsal thumb pressure in Figure 3 and Figure 4. It may indeed be more likely that the user intends to release when no pressure is applied, since most grasp epochs have already been directly labeled by palmar-direction pressure, and subjects are even less likely to press up against the exoskeleton to open their hands as opposed to when they intend to grasp. A potential improvement to the AC update assumption may be to make use of the task-specific prior distribution of grasp vs. release epochs (the distribution during training, for example). We could apply a Bayesian probability-based label to epochs without a clear intent from pressure, using information from both the classifier confidence as well as the prior distribution. 

Additional differences between the NA and AC controllers might be present in the surveys, motion capture data, and sEMG signals that were not analyzed for this paper, and is ongoing work. Further adaptation over time may also be examined in future work, as the system can still learn from the user during the sEMG-controlled phase. The behavior of the operator over time may also affect how the updates are made. If operators build trust in the system, they may expect specific exoskeleton behavior within a coordinated motion and pressure may again be minimal as the user waits for the exoskeleton to move. Since sEMG signals change with fatigue [36], this may also be a factor to consider for how the exoskeleton behaves over time. How and whether the exoskeleton’s control system converges to a correct user intent mapping over time may be an additional avenue of research, particularly in conjunction with changes due to fatigue and adaptation on the part of the user.

### 4.4. Anticipating the User

To our knowledge, this is the first experiment to utilize anticipatory sEMG signaling to improve the behavior of an upper-extremity exoskeleton controller. Anticipating the behavior of the upper extremities is more challenging than doing the same for the lower extremities, which typically see more regular and periodic motion. While other examples of sEMG-based exoskeleton control exist for the upper limbs (e.g., [3,18,30]), these methods rely on expert sEMG placement and do not use the sEMG signals to anticipate the human movement. Our results indicate that the trained sEMG-based classifiers are able to anticipate the user’s intent to grasp and initiate movement prior to a pressure-based system initiation. This particular part of the experiment built on the results of Beckers et al. [25] and Siu et al. [24], which showed the existence and usability of anticipatory sEMG signals in grasp and release. Although the difference in actuation timing is small, it could potentially reduce feedback control lag and alleviate some of the feeling of “sluggishness” that accompanies the reactionary control mechanisms used by typical force-based control [2]. Improving human-robot fluency in this manner is perhaps most important for augmentative exoskeletons, where the user is healthy and would have lower tolerance for system lag, as they are more likely to wear the exoskeleton for extended periods of time to increase strength or reduce injury risk.

### 4.5. Generalizability

Although we present an instance of the ILEXOS system for a thumb exoskeleton that used a supervised KNN classifier, the interaction-based learning framework of ILEXOS is agnostic to the nature of the exoskeleton actuation, as well as to the machine learning method used. To that effect, there are several generalizable advantages of the ILEXOS framework. 

First, the exoskeleton is usable while it is training as long as a simple intent mapping is obtained (e.g., pressure sensors, switches, inertial measurement, etc.). In contrast to systems that require the machine learning algorithms to explore the feature space by random perturbation [17,18], our use of a pressure-based initialization of the sEMG mapping allows us to avoid such perturbations. This removes additional “fitting” time of the exoskeleton, as well as any potential discomfort or safety concerns associated with random perturbation, particularly for exoskeletons with many powered degrees of freedom. While we do not necessarily explore as much of the feature space since we allow the users to select their own motions rather than have random perturbations, our procedure allows us to focus more specifically on the usable operating feature space. Additionally, we do not require a pre-built sEMG mapping as a starting point, as was done in [26], as ILEXOS learns each person’s mapping during training. This system behaves in the same way that many existing exosystems do (inferring intent based on pressure) during the period needed to train it, but post-training sEMG control offers a more coordinated and potentially less fatiguing control method. The latter is able to anticipate behavior and provide assistance without the additional applied forces required in a reactionary control.

Second, the system allows novice sensor placement. Non-specific placement of sensors is likely to be the norm with operational systems, and our system is shown to be robust to such placement, in addition to subdermal muscle shifts that are typical of a pick and place task. Allowing novice sensor placement is an important difference to note in comparison to the sEMG-controlled systems developed by Hamaya [17,18] and Kiguchi [26]. Our sensor placement was non-specific and over smaller muscle groups than the ones used in these two studies, which focused on gross arm movements.

Third, the system has the potential to improve over time, as additional data are collected from both the pressure and the sEMG sensors during the sEMG-controlled phase. The aforementioned modifications to the AC update assumptions may be necessary to ensure that times when the exoskeleton and the user are moving together fluently are labeled correctly, and we are able to avoid creating the strong bias towards a particular label that was observed in two of the subjects. The effect of different assumptions and timings for these updates should be considered in future work.

Since we tested ILEXOS using a discrete classifier with two states, our results are limited to considering operation in those states for the case of the book-placing task. The interactive learning-from-demonstration framework could be extended to a continuous control system, but that is not evaluated in the present study.

The system is generally applicable to different numbers of sensors and features. This study used six sEMG sensors, and fourteen sEMG features, but it does not specifically depend on any of these. Preliminary experiments have shown that intent recognition accuracy does not seem to suffer when decreasing the number of sensors and features, so further work could use a smaller set of sensors and features to reduce cost and computation time. However, it should be noted that as the number of sensors decreases, the precision with which the sensors must be placed would need to increase, since less of the forearm will be covered by sensors, presenting a trade-off that must be considered.

We also note that while supervised KNN is a fairly simple algorithm for nonlinear feature inputs like the present sEMG features, the algorithm has a linear time and space complexity of O(nd) for n observations with d dimensions, making it inappropriate to use the entire observed history when the training period is long. Subsampling, “forgetting” observations, using fewer features or a different choice of algorithm may yield improvements for long training times or cases of continuous updating. Additional work may attempt to find a better balance between values of n and d, since using fewer features could allow a longer observational history within the KNN model. It should be noted that support vector machine (SVM) approaches are commonly used for sEMG classification (e.g., [3], [19], [21], [30]) and do not suffer from increasing time complexity with the number of data points. Runtime for an SVM is linear with respect to the number of support vectors rather than the number of data points. A KNN method was chosen over SVM for this work due to higher accuracy during offline pilot testing using the amount of data that would be collected during a relatively short training period. Replacing the KNN with an SVM, or any other classification method, would not alter the conceptual adaptation methodology, but would affect the AC controller update process. Future work could examine the effects of changing the machine learning algorithm on the human-exoskeleton performance with ILEXOS.

### 4.6. Implications for Augmentative, Assistive, and Rehabilitative Exoskeletons

We motivated how the robust and anticipatory nature of our method has potential to improve the fluency for human-exoskeleton interaction in the augmentative case. The effects of the different controllers on human-exoskeleton fluency measures using motion capture and survey data collected during this experiment is ongoing work. The desire for improved fluency in the assistive case, where the exoskeleton aids in the movement of a non-healthy individual but is not doing so in a rehabilitative context is also of interest. For assistive exoskeletons, the robustness of our method to sensor placement may mean greater ease of use for at-home use cases. However, additional complexities arise when considering the assistive and rehabilitative use case. 

Progressive robot-assisted therapy, such as those using the wearable MyoPro exoskeleton [37] or the MIT Manus manipulandum [38] typically use muscle-specific sensors and threshold-based activation to encourage the association between certain muscle activations and a kinematic response in an assist-as-needed framework. There are several potential issues with using ILEXOS, and LfD in general, for a rehabilitative exoskeleton. First, it may be difficult or impossible for patients to train the system because they are unable to perform the requisite motions. This may be addressed by allowing subject intentions to be explicitly communicated via a switch, rather than inferred via pressure, while the patient attempts the action. Supervised labeling of intention may be most applicable in cases where the underlying sEMG signal is a weaker version of the normal signal, such as in multiple sclerosis [39]. In contrast, pathologies that produce other abnormal sEMG activations, such as stroke, may result in significantly lower classification accuracy [40]. The second concern relates to using an LfD mapping within therapy. While progressive therapy may be possible with the ILEXOS LfD framework by gradually increasing the confidence threshold needed to activate a particular action, the use of a personalized classifier may be problematic. The LfD mapping that makes ILEXOS easier for a healthy person to use may hinder rehabilitation as the users are encouraged to continue expressing the correctly classified sEMG signals that are nonetheless inappropriate for movement without the exoskeleton. This concern may be slightly alleviated by placing sEMG sensors on specific muscle bodies, but the concern remains that a well-mapped LfD system may be actuating with sEMG signals that are not conducive to rehabilitation. A model of appropriate changes in sEMG over the course of rehabilitation, or re-learning sEMG models in staged intervals may be necessary for a rehabilitation context. Further work is required to better understand the behavior of this kind of learning exoskeleton with patients undergoing rehabilitation, and how it may affect the rehabilitation process.

## 5. Conclusions

This paper presented a learning from demonstration exoskeleton system that transitions from a reactive pressure-based limit switch controller that is common in the current state of the art to an anticipatory sEMG-based controller. We show that this sEMG-based method is robust to novice sensor placement and subdermal muscle shifts during movement. The exoskeleton controllers show similar performance to a limit switch controller in terms of true positive grasp counts and times, but are able to move in time with the users’ thumbs by anticipating their actions approximately 0.2 to 0.3 s ahead of the pressure trigger. This learning from demonstration method has broader applicability to increasing the fluency of exoskeleton control by creating personalized sEMG mappings for individuals, and may also be applied to more complex multi-DOF exoskeletons without the need for perturbation-based system identification.

## Figures and Tables

**Table 1:** Subject characteristics.

Characteristic	Min	Max	Mean	Standard Deviation
Age (years)	18	33	22.5	4.5
Forearm Circumference (cm)	16	28	22.8	3.2
Seated Shoulder Height (cm)	46	66	55.6	5.6
Thumb Length (cm)	5	13	9.4	1.6
